# A quantitative comparison of different methods to detect cardiorespiratory coordination during night-time sleep

**DOI:** 10.1186/1475-925X-3-44

**Published:** 2004-11-25

**Authors:** Dirk Cysarz, Henrik Bettermann, Silke Lange, Daniel Geue, Peter van Leeuwen

**Affiliations:** 1 Department of Clinical Research, Gemeinschaftskrankenhaus Herdecke D-58313 Herdecke, Germany; 2 Institute of Mathematics, University of Witten/Herdecke D-58455 Witten, Germany; 3 Department of Biomagnetism, Research and Development Center for Microtherapy (EFMT) D-44799 Bochum, Germany

## Abstract

**Background:**

The univariate approaches used to analyze heart rate variability have recently been extended by several bivariate approaches with respect to cardiorespiratory coordination. Some approaches are explicitly based on mathematical models which investigate the synchronization between weakly coupled complex systems. Others use an heuristic approach, i.e. characteristic features of both time series, to develop appropriate bivariate methods.

**Objective:**

In this study six different methods used to analyze cardiorespiratory coordination have been quantitatively compared with respect to their performance (no. of sequences with cardiorespiratory coordination, no. of heart beats coordinated with respiration). Five of these approaches have been suggested in the recent literature whereas one method originates from older studies.

**Results:**

The methods were applied to the simultaneous recordings of an electrocardiogram and a respiratory trace of 20 healthy subjects during night-time sleep from 0:00 to 6:00. The best temporal resolution and the highest number of coordinated heart beats were obtained with the analysis of 'Phase Recurrences'. Apart from the oldest method, all methods showed similar qualitative results although the quantities varied between the different approaches. In contrast, the oldest method detected considerably fewer coordinated heart beats since it only used part of the maximum amount of information available in each recording.

**Conclusions:**

The method of 'Phase Recurrences' should be the method of choice for the detection of cardiorespiratory coordination since it offers the best temporal resolution and the highest number of coordinated sequences and heart beats. Excluding the oldest method, the results of the heuristic approaches may also be interpreted in terms of the mathematical models.

## Background

The time intervals between successive heartbeats (e.g. the RR tachogram or, equivalently, the series of instantaneous heart rates) may be analyzed with different tools to obtain information about e.g. heart rate variability (HRV) [1], regularity of the time series [2-7], or large-scale correlations in the time series [8-10]. Each technique provides different information about the 'time-structure' contained in the series of successive heartbeats. Some of them, like information obtained from HRV, may be linked to the sympathetic and parasympathetic activity of the autonomic nervous system [1,11]. Hence, they offer the possibility to interpret the derived quantities in terms of physiology. Furthermore, when applied to data obtained from patients suffering from heart diseases, these different information may be combined and used for risk stratification [12,13]. A large body of work concentrates on these univariate time series analysis and standards have been established [1].

To gain information that cannot be extracted from a single time series, the interaction between two (or more) physiological (sub-)systems has been investigated. This is often done on the basis of simultaneously recorded time series of each system. Hence, new bivariate techniques have been developed that aim to quantify the imprint of the physiological interaction between the two systems [14-17]. Especially, techniques which analyze the cardiorespiratory interaction have been developed recently [18-24]. Among these techniques the coordination between the series of successive heartbeats and the series of successive respiratory cycles, further denoted as cardiorespiratory coordination, is one focus of attention.

In fact, physiologists had already investigated cardiorespiratory coordination in the human organism as early as the 1960ies [25]. Calculating the distance between an inspiratory onset and its preceding R-peak they found intermittent coordination between heartbeat and respiration. In the 1970ies this interesting topic was no longer followed up (except in one study [26]), presumably because the physiological interpretation of the results was limited, although the last reviews of this era appeared in the late 1980ies [27-29]. The investigation of cardiorespiratory coordination has recently been revived mainly by physicists and mathematicians. They first studied the interaction of two weakly coupled chaotic oscillators and found synchronization of the phases whereas the amplitudes of the oscillators remained uncorrelated [30]. This class of mathematical models may serve as an approximate qualitative model for the interaction of heartbeat and respiration [19]. On the basis of these models new techniques for the analysis of bivariate time series data recorded from these physiological systems have been developed [22,31,32]. Other approaches have also been used to develop methods to analyze the cardiorespiratory interaction since the significance of this topic was deemed high [23,33-41].

The similarities or differences between the definitions of the different techniques and the advantages of each technique are not known yet. Unfortunately, a complete review and a (qualitative and quantitative) comparison of the recent literature is too extensive and beyond the scope of this paper. This study is restricted to the comparison of the performance of six different techniques in the detection of cardiorespiratory coordination. Although the comparison is limited, the insight that is drawn from it provides valuable information with regard to techniques that are not considered in this comparison. A second aim of this study is to investigate the cardiorespiratory interaction during night-time sleep since previous studies only dealt with relatively short recordings (no more than one hour).

## Methods

### Data acquisition and pre-processing

The electrocardiogram (ECG, standard lead II) and the uncalibrated nasal airflow (derived by a thermistor-technique) were recorded simultaneously in 20 healthy subjects (7 female, median age: 34.9 years, interquartile range: 13.7 years) using an ambulatory device (Medikorder, Tom-Signal, Graz). In each subject the night-time sleeping period was recorded, in some subjects the complete evening before the sleep period was also recorded. Hence, the total recording time varied from 7–16 hours. The device's internal sampling rate of the ECG was 3000 Hz. Thus, the times of the automatically identified R-peaks had an accuracy <1 ms and were written into a file. To save memory, the ECG was recorded using a rate of 250 Hz. The data were transferred to a PC and analyzed using Matlab (The Mathworks, Natick, Mass, USA) and C routines. The times of the automatically identified R-peaks were visually controlled, i.e. the times of the R-waves were marked in the ECG, and edited if the times of the automatically identified R-peaks did not match the R-peak in the ECG (<0.1% of all R-peaks). The times of the edited R-peaks had an accuracy of 4 ms because the recorded ECG had a lower sampling rate. Ectopies and artefacts were marked and excluded from the analysis.

**Figure 1:**
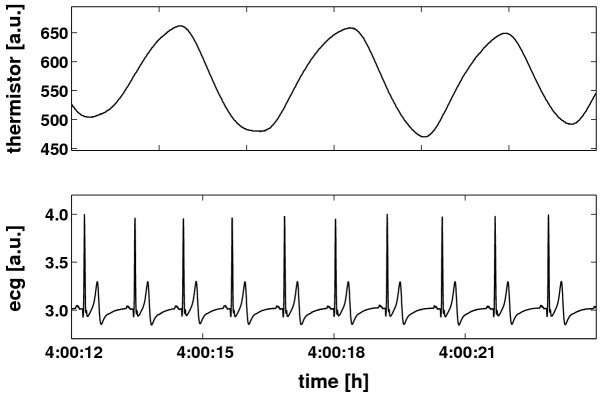
**Example of the recordings. **Example of a short sequence of the simultaneously recorded electrocardiogram (ecg) and respiratory trace (thermistor) during night time sleep.

**Figure 2:**
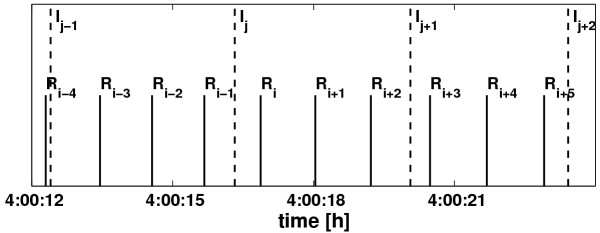
**Relevant data in the recordings. **The times of the R-peaks *R*_*i *_and the times of the inspiratory onsets *I*_*j *_(defined as local minima in the respiratory trace) derived from the recordings serve as event markers for further calculations.

The device's sampling rate of the nasal airflow was 100 Hz. The respiratory trace was saved into a file. Low frequency baseline trends were avoided using an internal filter with a 0.01 Hz cut-off frequency. The trace did not need any further pre-processing or filtering because it was smooth (cf. example in Figure 1). Inspiratory onsets were defined as local minima in the respiratory trace since local minima in the respiratory trace are due to the change from exhaling warm air to inhaling colder environmental air. They were extracted with an accuracy of 10 ms. For further analysis only the times of R-waves *R*_*i *_(*i *= 1,...,*n*_*R*_) and the times of the inspiratory onsets *I*_*j *_(*j *= 1,...,*n*_*I*_) were necessary and saved into a file. These data served as the basis for further calculations, see Figure 2.

Note that in the following the term 'cardiorespiratory coordination' is used descriptively in the sense that the times of the R-waves and the times of the inspiratory onsets show some kind of temporal incidence. The temporal incidence does not imply a physiological coupling between heartbeat and respiration, i.e. 'cardiorespiratory synchronization'.

The first step towards the analysis of cardiorespiratory coordination with respect to different *m:n*-coordination ratios (*m*: number of heart beats, *n*: number of respiratory cycles) is the calculation of temporal distances between the event markers of the two time series. Generally, two different useful possibilities have to be considered. (1) The temporal distance between inspiratory onsets and successive R-peaks, or, (2) the temporal distance between an inspiratory onset and the preceding R-peak.

1. Temporal distance between inspiratory onsets and successive R-peaks:

absolute distance: *t*_*i *_= *R*_*i *_- *I*_*j *_    (1)



2. temporal distance between an inspiratory onset and the preceding R-peak:

absolute distance: *t*_*j *_= *I*_*j *_- *R*_*i*-1 _    (3)



Note that for the calculation of the absolute distances *t*_*i *_and *n *> 1 the appropriate settings of the indices *i *and *j *have to be considered. Furthermore, the relative distance *φ*_*i *_corresponds to a definition of a phase angle of an oscillator on the interval [0, *n*]. This phase (multiplied by 2*π*) is equivalent to the cyclic phase calculated via the Hilbert-transformation [32,42]. Similar phases may also be calculated using e.g. the Fourier-transformation [43]. Hence, with respect to the variable *φ*_*i*_, the terms 'relative distance' and 'phase' are used synonymously throughout this paper. Compared to *φ*_*i *_and *t*_*i *_the calculation of *t*_*j *_and *β*_*j *_only needs the timings of the R-peaks before and after the inspiratory onset. Thus, these variables contain less information. They have been mainly used in early studies on cardiorespiratory coordination and are not common at present [44,45]. In order to keep this comparison practicable, the calculation of the absolute distances *t*_*j *_and the subsequent analysis of these quantities is left out in this study.

**Figure 3:**
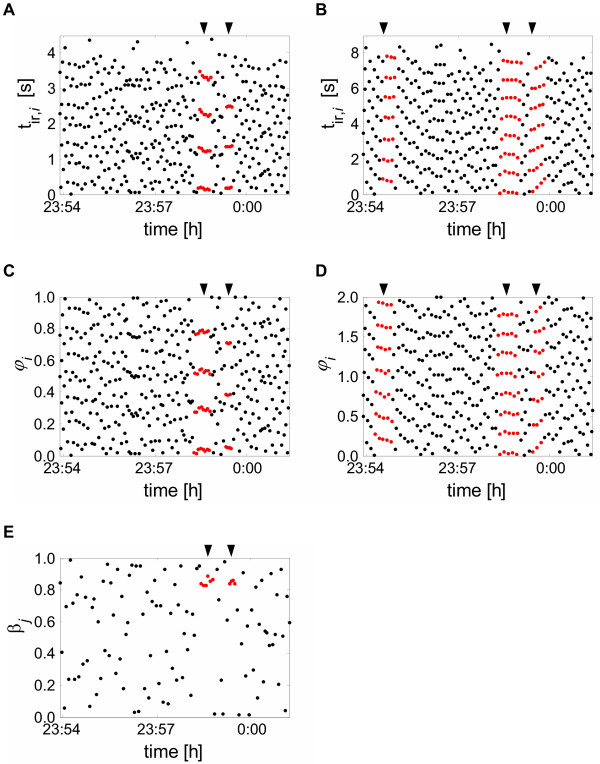
**Example of cardiorespiratory coordination. **(A) and (B) show examples of a 'Post Event Time Series' for 1 and 2 respiratory cycles, respectively. The corresponding 'Synchrograms' are shown in (C) and (D). (E) depicts the 'Synchrogram' for phases *β*_*j*_. In (A) and (C) the arrows indicate a sequence with 4:1- and a sequence with 3:1-coordination. In (B) and (D) the arrows point to sequences with 7:2-, 8:2- and 6:2-coordination. The arrows in (E) indicate sequences with coordinated inspiratory onsets that correspond to the coordinated sequences in (A) and (C).

In Figure 3 (a) an example of a succession of absolute distances *t*_*i *_is shown, i.e. each point represents the temporal distance of the R-peak to its preceding inspiratory onset (*n *= 1). In Figure 3 (b) these distances are calculated for two consecutive respiratory cycles (*n *= 2). This kind of representation is known as 'Post Event Time Series' [34,46] and has already been used in some early studies of cardiorespiratory coordination [26]. Since *n *= 1 in Figure 3 (a), the structures of parallel horizontal lines reveal 4:1- and 3:1-coordination (see marked points). In Figure 3 (b) the arrows indicate 7:2-, 8:2- and 6:2-coordination because *n *= 2 was used for the calculation of the distances. Figures 3 (c) and 3 (d) show the relative distances *φ*_*i *_of the same sequence for *n *= 1 and *n *= 2 respiratory cycles, respectively. This kind of representation is called 'Synchrogram' [19]. Obviously, it shows structures of parallel horizontal lines for the same epochs as the absolute distances *t*_*i *_(see markers in Figure 3). The structures with horizontal lines reveal epochs in which absolute distances *t*_*i *_or relative distances *φ*_*i*_, respectively, of each *m*-th R-peak recurs after one (*n *= 1) or two (*n *= 2) respiratory cycles: the cardiorespiratory interaction is coordinated. If the number of respiratory cycles *n *is increased (*n *> 2) these *m:n*-coordination may be observable in the same manner. In Figure 3 (e) the relative distances *β*_*j *_for the same sequence are shown. As mentioned above, this diagram contains less information compared to the other diagrams. Still, in the case of cardiorespiratory coordination, a short horizontal structure appears, cf. the markers in Figure 3 (e). These structures show the presence of *m:*1-coordination, similar to the diagrams in Figure 3 (a) and 3 (c).

The fact that cardiorespiratory coordination is present if structures with horizontal lines occur leads to the task of detecting these structures in the different diagrams. This task can be carried out by different techniques based on mathematical models or heuristic approaches. Some relevant approaches are described in the following.

### Detection of horizontal structures in Synchrograms or Post Event Time Series

#### 1. Detection via 'Synchronization-*λ*'

Along with the development of the Synchrogram a quantification on the basis of a mathematical model has been proposed [32,47,48]. Consider two coupled oscillators with appropriately defined phases *φ*_1 _and *φ*_2 _that may intermittently show a 1:1-synchronization (for simplicity both phases are defined on the interval [0, 2*π*]). In the synchronized case the difference *φ*_1 _- *φ*_2 _is constant. In real world data the phases *φ*_1 _and *φ*_2 _are contaminated by noise. Thus, even during synchronization the difference *φ*_1 _- *φ*_2 _is not constant. Rather it fluctuates around a constant. To take these influences into account a 'stroboscopic technique' has proven to be useful. Each time the phase *φ*_1 _exceeds a pre-defined value *θ *the phase *φ*_2 _is registered. During a synchronization the different values of *φ*_2 _are almost constant whereas during complete de-synchronization *φ*_2 _spreads equally over the interval [0, 2*π*]. This approach may easily be generalized for *m:n*-synchronization if the phases *φ*_1 _and *φ*_2 _are replaced by *φ*_1_/*m *and *φ*_2_/*n*. The distribution of *φ*_2 _may be quantified by the first Fourier mode.

The formalization of this idea is as follows: Observe the phase *φ*_2 _at the times *t *the phase *φ*_1 _is *φ*_1_= *θ *:



Next, calculate the first Fourier mode of the distribution of *ξ*_*i *_which is known as circular variance [49] (*M *values of *φ*_2 _have been observed):



If both oscillators are synchronized *λ *= 1, the completely de-synchronized case yields *λ *= 0. To get a more reliable result, *λ *may be calculated for different values of *θ *and subsequently averaged.

This method is easily applicable to the detection of cardiorespiratory coordination. Define the phases *φ*_1,2 _as follows: *φ*_1 _increases linearly on the interval [0, 2*π*] between two successive R-peaks and *φ*_2 _between two successive inspiratory onsets, respectively. To detect coordination for different *n:m*-ratios this method has to be applied for each desired *m:n*-ratio. In practice, coordination is present if *λ *exceeds a pre-defined threshold *λ *≥ *thres*_*λ*_. In this case the respective R-peaks are marked as coordinated.

Note that although this method deals with synchronization of two coupled oscillators it is not able to detect cardiorespiratory *synchronization *in a physical sense. A detection of synchronization on the basis of two simultaneously recorded time series would require supplementary information, e.g. the variation of the strength of coupling. Such supplementary information is not available for the cardiorespiratory interaction. Thus, the term cardiorespiratory *coordination *denotes a temporal incidence of events in both time series without claiming that this incidence is based on a coupling between both systems.

In this study, the window length *M *is set to *M *= 20. To obtain a better time resolution of the *λ*-values the window is forwarded one R-peak or accompanying phase, respectively. To obtain more stable values of *λ *the average of 10 different values of *θ*, equally distributed over the interval [0, 2*π*], is used. The threshold is set to *thres*_*λ *_= 0.85. This procedure is successively carried out for the following *m:n*-coordinations: for *n *= 1: *m *= 2,...,8 and for *n *= 2: *m *= 5,7,9,11,13,15.

#### 2. Detection via 'Phase Recurrences'

This method is based on a heuristic approach. Consider a synchrogram containing a sequence with *m *parallel horizontal lines indicating a *m:n*-coordination. In this sequence the relative distance *φ*_*i *_of each *m*-th R-peak has to be approximately the same. Otherwise parallel horizontal lines would not appear. This 'recurrence of phases' may be used to identify coordinated sequences [36] (a slightly different approach is presented in [50]).

The formalization of this concept is straightforward. For a *m:n*-coordination check whether the phase difference between the phase of R-peak *i *+ *m *and the phase of R-peak *i *is within a pre-defined tolerance *ε*. This condition has to be fulfilled for at least *k *successive R-peaks:



*N*_*r *_is the total number of R-peaks. In principle, the parameter k is not pre-defined. But, to be compatible with the description of 'parallel horizontal lines' during coordination, *k *≥ *m *needs to be fulfilled. This procedure allows a detection of a structure of parallel horizontal lines already with a length of 2*m *successive relative distances *φ*_*i*_. E.g. a 4:1-coordination may be identified already in a minimal sequence of 8 R-peaks. In the case coordination is detected, the respective R-peaks are marked as coordinated. To detect horizontal structures for different *m:n*-ratios this method has to be applied for each ratio. It is easy to implement and it can be used for absolute distances *t*_*i *_analogously.

In this paper the Phase Recurrence is analyzed with respect to absolute distances *t*_*i *_and relative distances *φ*_*i *_for the following *m:n*-coordinations: *n = *1: *m *= 2,...,8 and *n *= 2: *m *= 5,7,9,11,13,15. In the case of absolute distances *t*_*i *_the tolerance *ε *is set to *ε *= 0.075, i.e. 75 ms, and in the case of relative distances *φ*_*i *_the tolerance is set to *ε *= 0.025.

#### 3. Detection via 'Quantification of Histograms'

Another technique to detect cardiorespiratory coordination makes use of a sliding window comprising *n*_*F *_successive phases *φ*_*i *_that is moved over the entire series of phases [34,46]. First, a distribution of the phases *φ*_*i *_is calculated for each window. If cardiorespiratory coordination, i.e. a structure with parallel horizontal lines, is present the distribution of phases *φ*_*i *_shows some distinct equidistant local maxima (e.g. four local maxima in the case of 4:1 coordination). In the un-coordinated case the phases *φ*_*i *_are randomly distributed and local maxima do not appear. Next, each distribution is quantified by means of a Fourier Transformation. In the case of coordination, the distribution of the phases *φ*_*i *_contains several local maxima resulting in a huge maximum in the power spectrum. In the un-coordinated case the power spectrum does not contain any pronounced maximum since the phases *φ*_*i *_are equally distributed. Thus, the appearance of pronounced local maxima in the power spectrum is used to detect cardiorespiratory coordination.

This idea may be formalized as follows [46]. First, choose the length *n*_*F *_of the sliding window and the number of bins *k *to calculate the distribution. In the completely un-coordinated case the value of each bin *x*_*l *_is *n*_*F*_/*k*. If a perfect *m:n*-coordination is present the following distribution is obtained:



Here, *l*_0 _is the index of the bin that contains the first local maximum of the distribution. This distribution is quantified by means of a Fourier transformation:



The average of all bins *n*_*F*_/*k *is subtracted from each bin to avoid a dc-component in the power spectrum. In the un-coordinated case *P*_*x*_(*f*) = 0 for all frequencies *f*. In the case of a *m:n*-coordination *m *local maxima at the frequencies *f *= *a m*, *a *≤ *k*/(2*m*) appear. These local maxima are conserved even if the parallel horizontal lines are not exactly mapped into *m *bins but also some surrounding bins are filled. Thus, the distinction between coordinated and un-coordinated cardiorespiratory interaction may be carried out using the difference between the maximum and the minimum of the power spectrum.

*diff*(*P*_*x*_) = max(*P*_*x*_) - min(*P*_*x*_)     (10)

In the coordinated case this difference is large whereas in the un-coordinated case the difference is small. In practice, the R-peaks in the analyzed window are being marked as coordinated if *diff *(*P*_*x*_) exceeds a pre-defined threshold, i.e. if *diff *(*P*_*x*_) ≥ *thres*_*F*_. This procedure may analogously be applied to absolute distances *t*_*i*_.

Practically, as a compromise between the temporal resolution and a practicable estimation of the distribution, the window length is set *n*_*F *_= 20 and the window is forwarded 1 R-peak each time. The distribution has a bin-width of 0.1 sec for absolute distances *t*_*i *_and 0.025 for phases *φ*_*i*_, the number of bins *k *is adjusted accordingly. The threshold for identifying coordination is set to *thres*_*F *_= 12 for both, absolute distances *t*_*i *_and phases *φ*_*i*_. Notice that this implementation does not require a pre-selection of the integers *m *and *n *of a *m:n*-ratio.

#### 4. Detection via 'Quantification of the distribution of inspiratory onsets in RR-intervals'

In this study, the relative distances *β*_*j *_are also analyzed. They were used in the early studies of the 1960ies and in the subsequent studies of these research groups [25,27-29]. Although this technique does not use all the information available in the recording it is successfully used at present by one research group [44,51-53]. It has to be kept in mind that this technique yields coordinated inspiratory onsets instead of coordinated R-peaks. Thus, in order to get results which are comparable with the previously described techniques, the coordinated inspiratory onsets are replaced by R-peaks with the following rule: all R-peaks in a respiratory cycle that follow a coordinated inspiratory onset are marked as coordinated.

The quantification of the distribution of *β*_*j *_is carried out as follows. Since the values of *β*_*j *_are in the interval [0,1] they may be mapped onto the interval [0,2*π*] by multiplication with 2*π*. In this study, the distribution of *β*_*j *_in a data window of length *n*_*F *_is quantified by the calculation of the first Fourier mode of the distribution (the circular variance):



Analogously to the 'Synchronization-*λ*' defined above, the range of *γ *is 0 ≤ *γ *≤ 1. If *γ *= 0 *β*_*j *_is equally distributed indicating the completely de-coordinated case. If *γ *= 1 the *β*_*j *_is constant in the data window indicating the coordinated case. Practically, the inspiratory onsets in a data window are marked as being coordinated if *γ *≥ *thres*_*γ*_. Subsequently, the R-peaks in the respiratory cycle that follow a coordinated inspiratory onset are marked as coordinated. The window length *n*_*F *_is set to 10 and *thres*_*γ *_= 0.5. The data window is forwarded one inspiratory onset to achieve the maximal temporal resolution.

### Quantitative comparison of the methods, Statistics

**Figure 4:**
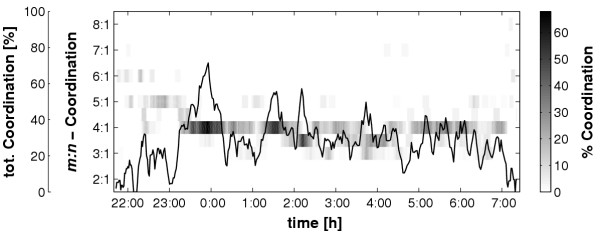
**Example of a 'coordination diagram'. **The 'coordination diagram' shows the course of the relative number of coordinated R-peaks (in a window of 500 R-peaks) at their respective *m:n*-ratio (method: 'Phase recurrences'). In this examples the 4:1-coordination prevails. The absolute maximum of 68% of coordinated R-peaks turns up at about midnight. The line in the diagram depicts the 'total coordination', i.e. the amount of coordination regardless of the *m:n*-ratio. Obviously, the cardiorespiratory coordination seems to oscillate.

The goal of the study was to compare quantitatively the results of the different techniques to analyze cardiorespiratory coordination. This was achieved in two steps. In the first step the cardiorespiratory coordination of each recording was analyzed with the following techniques: (1) 'Synchronization-*λ*', (2) 'Phase Recurrence' of relative distances *φ*_*i*_, (3) 'Phase Recurrence' of absolute distances *t*_*i*_, (4) 'Quantification of Histograms' of relative distances *φ*_*i*_, (5) 'Quantification of Histograms' of absolute distances *t*_*i*_, and (6) 'Quantification of Distribution of relative distances *β*_*j*_'. The detected R-peaks in horizontal structures, i.e. coordinated R-peaks, are marked with their corresponding number *m *of the *m:n*-ratio (e.g. all R-peaks in a sequence of 4:1 coordination were marked with 4). This allowed the distinction of coordinated R-peaks with respect to their *m:n*-ratio. Next, to reduce the amount of information, the percentage of coordinated R-peaks with a certain *m:n*-ratio in a window of 500 consecutive R-peaks was calculated. To get an adequate temporal resolution, the window was forwarded by 100 R-peaks until the entire series of R-peaks is covered. The percentage of coordinated R-peaks was coded as a greyscale plot because the percentage was plotted versus time for all analyzed *m:n*-ratios. This plot is called 'coordination diagram' (see Figure 4). In this diagram the amount of cardiorespiratory coordination and its respective *m:n*-ratio is easily accessible.

The total number of coordinated sequences and the total number of coordinated R-peaks during night-time sleep, i.e. from 0:00 to 6:00 assuming that all subjects were continuously asleep, served as quantitative measures of the performance of each method. They were calculated for each recording. Furthermore, the matrix of Pearson's correlation coefficients *r *between the coordination diagrams of the six methods was obtained as follows. Each correlation is calculated comparing the percentages of coordinated R-peaks with a *m:n*-ratio of two different methods for each data window. Furthermore, to enhance the validity of this comparison, only those pairs of percentages are used in which at least one percentage is greater than zero. Otherwise the large number of pairs with (0%,0%), i.e. the white area that both coordination diagrams have in common, would lead to a strong correlation even if the other parts of the coordination diagrams show apparent divergent *m:n*-coordination ratios.

## Results

An example of a 'coordination diagram' is shown in Figure 4. This example was analyzed with the method of 'Phase Recurrences' of relative distances *φ*_*i*_. Since the heart rate decreases during night-time sleep whereas the respiratory rate remains almost constant the *m:n*-ratios of cardiorespiratory coordination also decrease during this period. Thus the sleeping period may be estimated from the 'coordination diagram' by looking at the low *m:n*-ratios. In the example, the subject slept from 23:30 to 6:30 and had a predominant 4:1-coordination during night-time sleep. At midnight the maximum of approximately 68% is reached, i.e., 68% of all R-peaks in a window of 500 R-peaks showed a 4:1-coordination with respect to respiration. A second local maximum of 4:1-coordination is observable at 1:30. The rest of the night shows sequences with 4:1-, 7:2- (shown in between 4:1- and 3:1-coordination) and also some 3:1-coordination. Although not shown, it has to be added that qualitatively similar results were obtained with the other techniques. Furthermore, the analysis of the other subjects showed that the predominant *m:n*-coordination ratio during night-time sleep of each subject varies inter-individually and is one of the following ratios: 3:1, 4:1, 5:1, 7:2 or 9:2.

The additional line drawn in the coordination diagram represents the 'total coordination', i.e., the percentage of coordinated R-peaks in a window of 500 consecutive R-peaks regardless of the *m:n*-ratio. As indicated by the time course of the separate *m:n*-coordinations the total coordination is not constant or a simple function of time. Instead, the total coordination seems to oscillate. In the course of the night-time sleep the frequency of the oscillation increases whereas the amplitude decreases. Similar qualitative results were obtained using the other techniques.

The total number of coordinated R-peaks *N*_*R *_and the total number of coordinated sequences *N*_*seq *_during night-time sleep contain important quantitative information about the performance of the different methods (see Table 1). The group's median total number of coordinated R-peaks ranges from *N*_*R *_= 1673 R-peaks for the 'Quantification of inspiratory onsets in RR-intervals' (Hist *β*_*j*_) to *N*_*R *_= 6158 R-peaks for the detection via 'Phase Recurrences' of phases *φ*_*i *_(PhRec *φ*_*i*_), i.e. 7.7% to 30.4% of the total number of R-peaks during night-time sleep are coordinated with respiration. The 'Phase Recurrences' of times *t*_*i *_and the 'Quantification of Histograms' of phases *φ*_*i *_also detect a high number of coordinated R-peaks during night-time sleep (*N*_*R *_= 5465 and *N*_*R *_= 5146, respectively). Notice that the number of coordinated sequences is highest for the 'Phase Recurrences' (*N*_*seq *_= 416 for relative distances and *N*_*seq *_= 398 for absolute distances) compared to other methods. Hence, the coordinated sequences detected by the 'Phase Recurrences' are shorter than the sequences detected by other methods. Although not shown, it has to be added that the high number of coordinated sequences and R-peaks for the 'Phase Recurrences' is due to the setting *k *≥ *m *for equation (7). If this setting is changed to *k ≥ m *+ 8, i.e. the requirement |*φ*_*i *_- *φ*_*i*+*m*_| <*ω *has to be fulfilled at least *m *+ 8 times, the median number of coordinated epochs decreases to *N*_*seq *_= 206 and the median number of coordinated R-peaks decreases to *N*_*R *_= 4273.

**Table 1:** Number of detected sequences with cardiorespiratory coordination. Median and inter-quartile range during night-time sleep of the following parameters: *N*_*R *_total number of coordinated R-peaks, *N*_*seq *_total number of coordinated sequences, % *N*_*R *_relative amount of coordinated R-peaks with respect to the total number of R-peaks.

	Sync *λ*	PhRec *t*_*i*_	PhRec *φ*_*i*_	Hist *t*_*i*_	Hist *φ*_*i*_	Hist *β*_*j*_
*N*_*R*_	3134 (2957)	5465 (3776)	6158 (2696)	3502 (4818)	5146 (3795)	1673 (1288)
*N*_*seq*_	123 (98)	398 (266)	416 (126)	140 (176)	191 (118)	31 (21)
% *N*_*R*_	14.6 (11.2)	27.8 (14.0)	30.4 (10.8)	16.8 (17.6)	24.3 (12.0)	7.7 (5.9)

**Figure 5:**
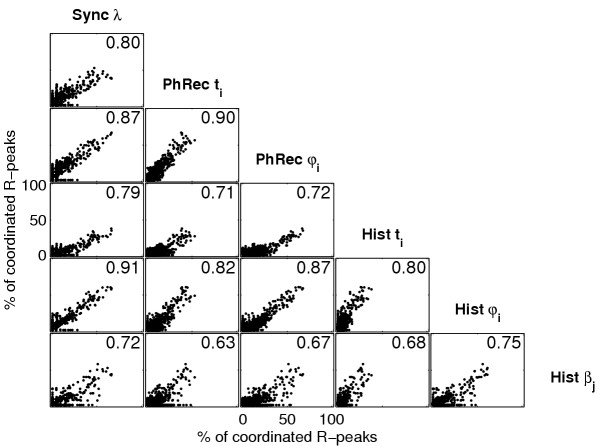
**Example of the correlations between the different methods. **Example of the correlations between the different methods to detect cardiorespiratory coordination.

An example of the correlation between the coordination diagrams of the different methods is shown in Figure 5. For this subject the correlation coefficient *r *ranges from *r *= 0.63 to *r *= 0.91 indicating a good agreement between the different coordination diagrams. The coordination diagrams for 'Synchronization-*λ*' and 'Quantification of Histograms' of relative distances *φ*_*i *_correlate strongest (*r *= 0.91). And also the coordination diagrams for 'Phase Recurrence' of absolute distances *t*_*i *_and 'Phase Recurrence' of relative distances *φ*_*i *_show a strong correlation (*r *= 0.90).

The matrix of correlation coefficients was calculated for each subject. Table 2 lists the median and the interquartile range of each item in the matrix. Again, the results of the analysis of the 'Synchronization-*λ*' and the 'Quantification of Histograms' of relative distances *φ*_*i *_correlate strongest (median correlation *r *= 0.89), followed by the correlation between the 'Phase Recurrences' and the 'Quantification of Histograms' of relative distances *φ*_*i *_(*r *= 0.82) and the correlation between the 'Phase Recurrences' of relative distances *φ*_*i *_and the 'Phase Recurrences' of absolute distances *t*_*i *_(*r *= 0.81). In all rows of the matrix except the last the correlation coefficients are *r *> 0.64 indicating a strong correlation between results of the different techniques. Since all correlation coefficients in the last row are *r *< 0.5 the correlation between the results of the 'Quantification of inspiratory onsets in RR-intervals' (Hist *β*_*j*_) and all other techniques is weaker.

**Table 2:** Correlations between the different methods to detect cardiorespiratory coordination. Median and inter-quartile range of the correlations coefficients between the different methods to detect cardiorespiratory coordination.

	Sync *λ*	PhRec *t*_*i*_	PhRec *φ*_*i*_	Hist *t*_*i*_	Hist *φ*_*i*_	Hist *β*_*j*_
Sync *λ*	1.00					
PhRec *t*_*i*_	0.66 (0.20)	1.00				
PhRec *φ*_*i*_	0.78 (0.18)	0.81 (0.10)	1.00			
Hist *t*_*i*_	0.60 (0.21)	0.75 (0.16)	0.70 (0.18)	1.00		
Hist *φ*_*i*_	0.89 (0.11)	0.72 (0.14)	0.82 (0.09)	0.64 (0.26)	1.00	
Hist *β*_*j*_	0.45 (0.36)	0.35 (0.28)	0.37 (0.30)	0.45 (0.34)	0.49 (0.28)	1.00

## Discussion

In recent years a variety of different methods have been proposed to detect cardiorespiratory interaction on the basis of simultaneously recorded time series of heart beat and respiration. In this study, six different methods to identify cardiorespiratory coordination based on mathematical models or heuristic approaches have been quantitatively compared. The main issue of the different methods is the recognition of coordination in synchrograms and post event time series, i.e. the detection of structures with horizontal lines in these representations. Thus, the procedures permit the quantification of qualitative information that is contained in these representations and may provide new information that is not extractable from the isolated univariate time series.

The quantitative comparison of the six different methods to detect cardiorespiratory coordination showed that the group's median total number of coordinated R-peaks and the median total number of coordinated sequences varied depending on the detection method used. The largest number of coordinated R-peaks (30.4% of all R-peaks during night-time sleep) and coordinated sequences was detected by the 'Phase Recurrences' of phases *φ*_*i*_. Since the coordinated R-peaks are distributed in the largest number of coordinated sequences this method has the best temporal resolution compared to all other methods used in this study. Furthermore, it is able to detect even very short and intermittent cardiorespiratory coordination because the implementation used in this study only required at least 2*m *R-peaks to detect a *m:n*-coordination (typically, *m *= 2,3,...,8). But, if the minimal number of required coordinated R-peaks is increased to e.g. 2(*m *+ 8), this method shows approximately the same number of coordinated epochs and R-peaks as e.g. the 'Quantification of Histograms'. Hence, if the 'window length' of this method is increased, the results are similar to those of the methods with a window length of 20 R-peaks. Since the 'Phase Recurrences' of absolute distances *t*_*i *_also showed a large number of coordinated R-peaks and coordinated sequences, respectively, and the correlation to the results of the 'Phase Recurrences' of phases *φ*_*i *_is strong, these procedures showed approximately the same characteristic features. All other methods were less sensitive in the detection of cardiorespiratory coordination because the identification of cardiorespiratory coordination required a minimal window length of 20 R-peaks. The method of 'Quantification of inspiratory onsets in RR-intervals' had an even longer window length, provided that the window length of 10 inspiratory onsets is equivalent to approximately 40 R-peaks since the average ratio of heart rate and respiratory rate, irregardless of any cardiorespiratory coordination, is 4 [35]. Thus, these methods are not as suitable for the detection of cardiorespiratory coordination as the method of 'Phase Recurrences'. Nevertheless, although all methods had different definitions and different implementation details, they revealed similar qualitative features of cardiorespiratory coordination during night-time sleep, i.e., the coordination diagrams showed approximately the same structure and the 'total coordination' (percentage of all coordinated R-peaks regardless of the *m:n*- ratio) was not constant but oscillated during night-time sleep. Since this oscillation correlates with heart rate variability (HRV) [36] and different sleep stages also correlate with HRV [54,55], this oscillation may have its origin in the sleep architecture.

Another quantitative comparison was carried out by intra-individually correlating the 'coordination diagrams' of each method. The correlation coefficients served as a measure of similarity between the different diagrams. The strongest correlation was observed between the coordination diagrams derived from the 'Synchronization-*λ*' and the 'Quantification of Histograms' of relative distances *φ*_*i*_. Furthermore, the 'Phase Recurrences' of relative distances *φ*_*i *_and of absolute distances *t*_*i *_correlated strongly. These results indicate that the amount of information contained in the relative distances *φ*_*i*_, i.e. the relative timings of the R-peaks in a full respiratory cycle, and in the absolute distances *t*_*i*_, i.e. the timings of R-peaks with regard to the preceding inspiratory onset, was approximately the same. Furthermore, the results of the 'Synchronization-*λ*' showed strong correlations to the 'Phase Recurrences' and the 'Quantification of Histograms'. Thus, the latter methods, which have heuristic origins, may also be explained mathematically by the mathematical models used to develop the method of 'Synchronization-*λ*'. However, the correlation coefficients concerning the 'Quantification of Histograms' of relative distances *β*_*j*_, i.e. the relative timing of an inspiratory onset in the respective RR-interval, were lower. Thus, these distances seem to contain different information. This difference is attributed to the fact that the calculation of the relative distances *β*_*j *_is not based on the full amount of information available in the recording.

Taken these findings together, it is apparent that the different methods may lead to similar results if the maximum amount of information is used. It is important to keep in mind that all quantifications are based on the 'Post Event Time Series' or the 'Synchrogram'. Thus, although some methods have heuristic origins and some have a mathematical model as its origin and although the different approaches differ considerably with respect to their calculations, the relevant information of the 'Post Event Time Series' and the 'Synchrogram' is captured by the different methods.

A debate exits whether the cardiorespiratory coordination is due to physiological interaction between the involved systems or other mechanisms might be responsible, like e.g. a reduced variability in at least one of the involved systems [36,56]. Surrogate data have been used to give at least a quantitative answer to this debate [34,38,57]. The analysis of surrogate data suggests that cardiorespiratory coordination is sometimes due to some kind of physiological interaction. Another approach that may be of importance is the stochastic phase synchronization, i.e. the synchronization between coupled systems due to stochastic stimuli [58,59]. However, this kind of mechanism has not been explored for the cardiorespiratory system and the implications are not clear yet. Irrespective of this debate, it is an intriguing phenomenon that heart rate and respiratory frequency show a 4:1-ratio on average during night-time sleep [35,60]. The present results also indicate that there seem to be some constraints with respect to variability and other features of the involved systems to achieve certain *m:n*-ratios. Further methods and models have to be developed to explore these features of the cardiorespiratory system.

In conclusion, the different methods to detect cardiorespiratory coordination show similar qualitative results. The method 'Phase Recurrences' has the ability to detect most sequences of heart beats coordinated with respiration. It is able to maximize the temporal resolution because even very short sequences may be detected. Thus, this method should be preferred. The method of 'Quantification of Histograms' of relative distances *β*_*j *_contains different quantitative information since only parts of the full amount of information available is used. Hence, this method is not recommended because a comparison with the other methods is limited. Generally, using appropriate methods, cardiorespiratory coordination is detectable in approximately 20–25% of all heart beats during night-time sleep. Future work should focus on the prerequisites of the appearance of cardiorespiratory coordination during night-time sleep and whether this appearance is linked to sleep stages. Furthermore, a preliminary study has shown the loss of cardiorespiratory coordination in patients after acute myocardial infarction [61,62]. Hence, the gain of prognostic information of this bivariate analysis should be explored further.

## Authors' contributions

DC and HB designed the study, recruited the subjects and collected the data. DC carried out the analysis and drafted the manuscript. SL, DG and PvL were involved in the interpretation of the data and participated in the final revision.
